# A Case Report of Median Nerve Entrapment in a Supracondylar Humeral Fracture: Diagnosis, Treatment, and Results After 5 Years of Follow-Up

**DOI:** 10.3390/reports8010023

**Published:** 2025-02-18

**Authors:** Carlo Colonna, Joil Ramazzotti, Francesco Locatelli, Alessandro Crosio, Pierluigi Tos

**Affiliations:** 1 ASST Papa Giovanni XXIII, 24127 Bergamo, BG, Italy; 2 ASST Bergamo Ovest, Treviglio Hospital, 24047 Treviglio, BG, Italy; joil.ramazzotti3449@gmail.com; 3 Hand Surgery and Reconstructive Microsurgery Department, ASST Gaetano Pini-CTO, 20122 Milan, MI, Italy; francescomaria.locatelli@asst-pini-cto.it (F.L.); alessandro.crosio@gmail.com (A.C.); pierluigi.tos@unito.it (P.T.)

**Keywords:** median nerve palsy, supracondylar fracture, Gartland type III, US

## Abstract

**Background and Clinical Significance**: Neurological complications in extension-type-III supracondylar humeral fractures (SCHFs) in children represent 11% of cases. An extension-type-III SCHF with posterolateral displacement of the distal fragment is commonly associated with damage to the median nerve and the anterior interosseous nerve (AIN). Neurological complications are often unnoticed, and their immediate postoperative diagnosis is difficult, particularly in young children. Neurapraxia, the most common complication, usually undergoes spontaneous nerve recovery. **Case Presentation**: We report a case of a 7-year-old patient with postoperative median nerve palsy after an SCHF (Gartland type III) who was referred to our unit from another hospital due to a lack of spontaneous recovery. In addition, motor and sensory functions were absent. As ultrasound (US) indicated nerve kinking at the fracture site, an exploration was performed. The nerve was trapped within the fracture and the callus. It was surgically extracted, and intraoperative examination with US indicated that resecting the kinked nerve, freeing the two stumps, and attempting a primary end-to-end suture represented the best course of action. We present this case with a 5-year follow-up surgery, which showed a good clinical outcome. **Conclusions**: This case is noteworthy because of its diagnostic and therapeutic pathways, and it is complemented by surgical and ultrasound images that can assist other surgeons in similar circumstances.

## 1. Introduction and Clinical Significance

Neurological complications in extension-type-III SCHFs in children represent 11% of cases with complete median nerve or isolated anterior interosseous nerve (AIN) injuries, which account for over 60% of neurological injuries [1,2,3,4,5]. The vulnerability of the anterior interosseous nerve may be attributed to the anatomical positioning of its exclusively motor posterior fascicular structures within the median nerve, making it more susceptible to injury or to extension into the proximal region of the forearm [6,7,8,9].

Other neurological complications aside from SCHFs include radial nerve injuries associated with extension-type fractures with posteromedial displacement (6–13.5%) and ulnar nerve injuries after flexion-type fractures (3–12%) [10]. The majority of nerve deficits related to extension-type SCHFs are neuroapraxic and recover spontaneously. However, in a minority of patients with more severe injuries of axonotmesis or neurotmesis, neurological functions fail to return without surgical intervention [11]. Approximately 1 to 19% of patients with supracondylar fractures present with an absent pulse; however, only a minority of these patients require vascular repair [12,13].

Despite the availability of various diagnostic tools (magnetic resonance imaging, ultrasound and electrodiagnostic testing) for studying acute traumatic nerve injuries, it is challenging to precisely interpret the results obtained from individual examinations and, therefore, to predict short- and long-term outcomes after trauma [2].

Regarding neurovascular complications, whether to use surgical or conservative approaches is still a matter of discussion [6,10,14,15].

A case of median nerve palsy was reported where the nerve was trapped in the SCHF during fracture reduction and fixation in a 7-year-old patient. Surgeons initially misdiagnosed median nerve paralysis; after six months with no improvement, a US examination indicated nerve entrapment in the fracture (EMG reported neurotmesis), while surgery revealed a complete nerve interruption with entrapment at the fracture site. A direct suture (end-to-end repair) without grafts was performed.

### Clinical Significance

This study highlights the potential severity and complexity of median nerve entrapment within a pediatric supracondylar humeral fracture and its subsequent fixation. Although neuropraxic injuries are common and often resolve spontaneously, this case demonstrates that more severe nerve injuries, such as a complete nerve trunk disruption (Sunderland IV), may occur. Early recognition through detailed clinical, electrophysiological, and imaging evaluations is critical. Definitive surgical exploration and nerve repair, even performed several months post-injury, can lead to meaningful functional recovery. This report underscores the importance of vigilance and timely intervention in cases of persistent nerve deficits following fracture management.

## 2. Case Presentation

**Figure 1:**
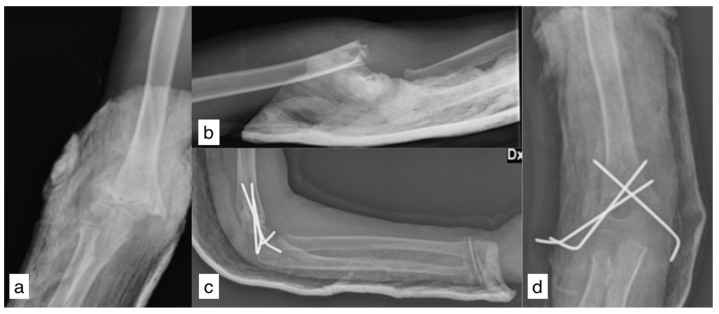
Pre-operative X-rays (**a**,**b**); postoperative X-rays (**c**,**d**).

A 7-year-old right-handed female patient fell from 5 m high with an outstretched right arm. The arm was primarily swollen with gross deformities, but no neurovascular impairment was found. X-rays showed a posterolateral dislocated Gartland-type-III SCH fracture (Figure 1a,b). A mini-open reduction through lateral incision and fixation with Kirchner wires was performed in another hospital. Postoperative X-rays showed a satisfactory reduction and no inter-fragment gap (Figure 1c,d).

Radiological fracture healing occurred in two months, allowing for pin removal to be performed. During hospitalization, no neurovascular impairments were noted.

One month later, the patient and the parents complained about dysesthesia in the index and thumb of the right hand, along with difficulty in moving those fingers. The mother also observed an altered profile in the daughter’s hand. The patient was referred to our hospital 5 months after the trauma *occurred while* waiting for spontaneous recovery.

Clinical examination *at 5* months revealed an inability to flex the terminal joints of the thumb and the distal interphalangeal joint (DIJ) of the index finger, as well as the flexor carpi radialis; a complete impairment of the thenar muscles; and hypoesthesia in the median nerve area with the absence of sweating—a representation of complete median nerve palsy. A Tinel sign test showed positive results, without distalization, at the level of the humeral fracture fixed 5 cm proximally to the elbow crease.

A US was the first exam requested to evaluate the nerve condition after EMG; it showed median nerve sufferance with peripheral edema (the maximum caliber was 24 mm) and a sudden interruption of its course at the volar margin of the humeral fracture. The entrapment at the fracture site was clear *and* no lesions of the brachial artery were detected. Electroneurography revealed delayed antidromic sensory conduction with prolonged latency of the median nerve between the second and third fingers. An MRI confirmed the sufferance of the forearm muscles innervated by the median nerve, but no clear picture of the nerve condition was observed in the arm.

**Figure 2:**
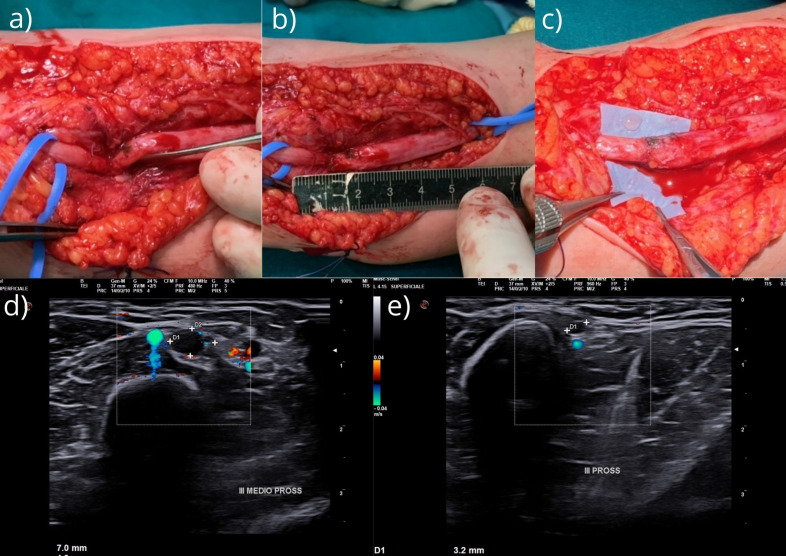
(**a**) Median nerve incarcerated in the fracture site; (**b**) extension of the median nerve glioma; and (**c**) end-to-end suture and epiperineural suture with 9–0. Furthermore, a stitch of 7–0 in the epineural part of the nerve was proximally and distally placed 2 cm from the suture site to discharge the tension at the suture site. Intraoperative US: (**d**) nerve sufferance with peripheral edema (the maximum caliber was 24 mm) and (**e**) a sudden reduction in nerve caliber and interruption of its course at the volar margin of the humeral fracture.

We opted for an immediate surgical exploration and treatment of the median nerve lesion at the fracture site; this was performed 6 months after the trauma occurred. Surgical exploration and dissection under the microscope allowed the nerve to be extracted from the bone callus and the fracture (Figure 2a); the nerve appeared to be completely kinked and incarcerated in the fracture callus. During careful dissection and excision of the scar tissue, the nerve was identified and released.

Intraoperative nerve stimulation did not show any activation of muscles innervated by the median nerve, but the intraoperative US revealed complete transections of the fascicular pattern; only the epineurium tissue exhibited continuity (Figure 2d,e). The funicular pattern was proximally and distally present 1 cm from the lesion. Neurotomy with excision of the distal glioma and proximal neuroma was performed under magnification, and scar tissue was removed with a progressive slicing technique for the “normal” funicular pattern. At the end of the neurotomy, the substance loss was 2.5 cm (Figure 2b). We then freed the proximal and distal stumps for a distance of 3–4 cm on both sides and were able to avoid using grafts via end-to-end suturing of the nerve (microscope and epiperineural suture with 9–0) while flexing the elbow 30° (Figure 2c). Furthermore, a stitch of 7–0 in the epineural part of the nerve was proximally and distally placed 2 cm from the suture site to discharge the tension at the suture site [16]. We added fibrin glue at the end of the suture. The elbow was immobilized by a posterior splint flexed at 90° to protect the suture from any tension for 3 weeks; this angle was then progressively extended by 20 degrees per week up to complete extension. Rehabilitation was performed by a hand physiotherapist.

A follow-up at one month showed no substantial difference. After six months, the Tinel sign test presented a positive result at 15 cm distal to the suture site. Partial motor recovery was noted through initial index distal phalanx flexion and improvement of the thumb–index pinch. Sensitivity returned completely and amyotrophy of the thenar eminence disappeared.

At the 12-month follow-up, a subtotal recovery of the median nerve function was observed with the increased strength of the thenar muscles (M4), flexor longus index (M4), and flexor longus pollicis (M4). The Tinel sign disappeared. Minor hyperalgesia has been noted as well as difficulty in using the upper limb during the patient’s altered perception of sensitivity.

At the 24-month follow-up, a complete recovery was noted: thenar muscles, flexor longus index, and flexor longus pollicis were graded as M5 after performing a 4 mm TPD, showing good and free use of the upper limb and hand.

**Figure 3:**
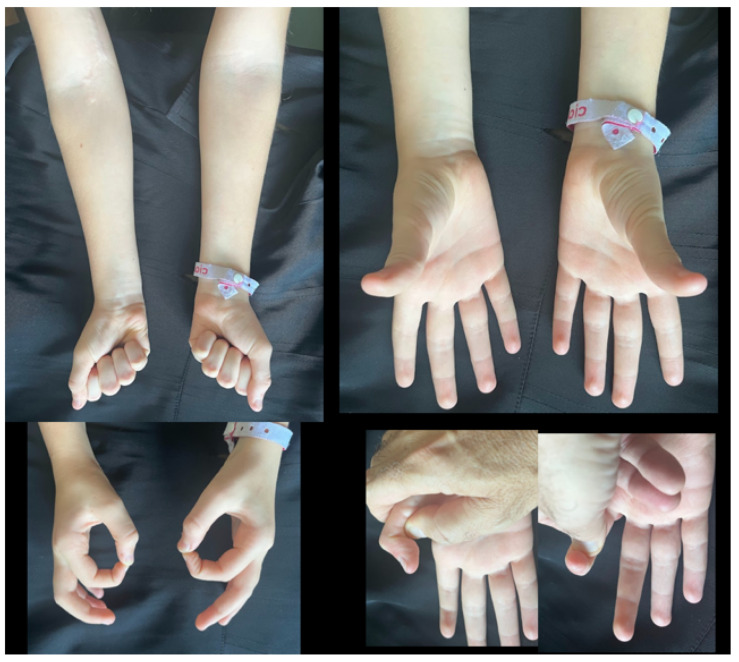
A five-year follow-up showing complete sensory–motor recovery of the right hand.

Five years later, clinical examination revealed that the patient had complete sensory–motor recovery of the right hand, returning to normal daily activities (Figure 3).

## 3. Discussion

Extension-type-III SCHFs with posterolateral displacements of distal fragments are commonly associated with damage to the median nerve and AIN [9,10,11,12]. In the early phases, neurological complications in younger children are often unnoticed [13], but these most often comprise neurapraxia, and spontaneous nerve recovery should be checked upon follow-up [11,17].

In 1986, J Karlsson et al. concluded that, when severe neurovascular disturbance is present and not improved through standard management, the brachial artery and median nerve should be examined without delay [15]. In a retrospective study, Gosens and Bongers found 11 cases of median nerve damage with up to 189 SCHFs treated using closed reduction and pin fixation (10 cases were treated using open reduction). All patients recovered without sequelae [18]. Louahem et al. reviewed 210 cases of severely displaced SCHFs and found that 13.5% of cases were complicated by median nerve injuries. AIN palsy was the most common complication, occurring in 18 cases, but it was only noted as a pre-operative diagnosis in half of the cases. In their research, injury to the AIN is always associated with posterolateral displacement. Almost all of the cases with neurological complications recovered spontaneously [6]. Median nerve entrapment after displaced SCHFs in children remains relatively uncommon but should not be underestimated.

It is important to immediately ascertain if there are any vasculonervous deficits after surgery. In the present case, proper clinical investigation was not carefully performed before and after fracture fixation.

When the problem is noticed, a US performed by an expert radiologist can quickly show the type of lesion [2] and indicate whether surgery should be performed immediately.

Shih Chien-An et al. explored the possibility of performing closed reduction and internal fixation procedures for pediatric distal humeral fractures (SCHFs) using ultrasound, demonstrating radiographic results comparable to those obtained with traditional methods and reducing the risk of iatrogenic ulnar nerve injuries [19]. Although pre-operative clinical examination is the primary diagnostic tool for identifying acute traumatic nerve injuries following SCHFs, further research could confirm the usefulness of intraoperative ultrasounds, in conjunction with X-rays, for studying the continuity of at-risk nerve structures and reducing the risk of iatrogenic injuries.

Felici et al. suggested nerve reconstruction with grafting when a closed lesion of the median nerve does not show spontaneous recovery within 6–12 months after trauma [20].

Luckily, nerve recovery after surgery in young patients is very good, and full recovery can be achieved even 3–4 months after the injury.

We also point out how, in these cases, it is possible, with a resection of 2–3 cm, to perform a direct suture that certainly gives better results than a suture with nerve grafts [21,22].

Regarding the length defect of the nerve, the direct end-to-end repair of the nerve injury currently represents the gold standard surgical approach for severe nerve injuries with a gap of less than 3 cm [23]. When primary repair cannot be carried out without excessive tension, interposition nerve grafting becomes necessary, with autografts being the preferred standard for grafting materials [24].

Unloading the suture with a 7–0 stitch and slight flexion of the elbow can improve the final result.

The complete sensory–motor recovery described in the study may be attributed to the patient’s young age, the limited gap between median nerve stumps, and a reasonable time interval between the traumatic event and nerve repair.

The patient should be referred to surgeons with microsurgical expertise who specialize in nerve repair at the earliest convenience. A few years ago, surgeons waited and performed surgical exploration only in cases of persistent neurological complications with no clinical or nerve conduction evidence of recovery at 3 months [8,10,25,26]. The advent of high-frequency US and radiologists with good experience in the field allowed for a better anatomopathological understanding of nerve injuries, especially when the nerve is severed or trapped in the fracture. If the US is not clear, re-examination at a later time is a good alternative; in addition, Tinel sign tests and progressive recovery can aid in recommending abstention or surgery.

We recognize the limitations of this study’s format as it is a case report; however, we emphasize the important role of specialized nerve surgeons and radiologists who can easily make anatomopathological diagnoses to identify the type of injury and guide pre-operative planning. Similarly, this study’s results provide recommendations for the surgical treatment of such injuries, which are not frequently described as a direct suture after a 2.5 cm nerve resection. This could be useful for other surgeons facing similar situations.

## 4. Conclusions

Median nerve entrapment, even if uncommon, must be considered a complication after a posterolateral displaced SCHF in children. Clinical suspicion is the first step in identifying neurological complications following a supracondylar humeral fracture in the pediatric population.

If this complication is not recognized and treated immediately, a combination of diagnostic tools like US, MRIs, and electrodiagnostic testing can determine the continuity of the nerve, the severity of the injury and the site in order to guide our treatment decisions.

US and expert radiologists specializing in nerves play a fundamental role in the pre-operative diagnosis of acute traumatic peripheral nerve injuries with closed skin. The use of an intraoperative ultrasound could represent valuable diagnostic support to further reduce the incidence of unrecognized or iatrogenic nerve injuries in the pre-operative phase. A direct suture is possible in that zone and in that situation.

This patient should be referred immediately to centers with high-expertise staff and where a microsurgery unit is present.

## Data Availability

The original contributions presented in this study are included in the article. Further inquiries can be directed to the corresponding author.
